# Lights, P450, action! Metabolite formation in chloroplasts

**DOI:** 10.1093/jxb/erw114

**Published:** 2016-03-23

**Authors:** Heribert Warzecha

**Affiliations:** Plant Biotechnology and Metabolic Engineering, Technische Universität Darmstadt, Schnittspahnstraße 3–5, 64285 Darmstadt, Germany

**Keywords:** Cytochrome P450, plastid transformation, metabolic engineering, plant natural products

**The capacity of plants to convert light into sugar and, consequently, to build specialized compounds of vast complexity is a well-established phenomenon. In their study in this issue of *Journal of Experimental Botany*, Gnanasekaran *et al.* (pages 2495–2506) show that the most versatile catalysts of specialized metabolism can be fueled directly by photosynthetic electron flow. This finding might have great implications for future plant metabolic engineering endeavors.**

Plants, being sessile organisms, rely on myriad chemicals as the means of interaction with their environment. Collectively referred to as ‘specialized compounds’, they comprise structurally variable metabolites from diverse classes, such as terpenoids, phenylpropanes and alkaloids, to name only a few major groups. Besides helping plants to attract pollinators or deter enemies, many of these compounds have become directly important to us. Applied as fragrances, flavors, fine chemicals or medicines, they are being extracted in large amounts, traded and widely utilized – they have been rightly dubbed ‘plant natural products’ (PNPs). Understanding the biosynthetic capacity behind specialized compound accumulation and exploiting this natural ‘warehouse’ has, therefore, become a major scientific goal. Ultimately, elucidation of metabolite formation will facilitate targeted engineering of pathways – modifying plants to spur production of compounds of interest in defined amounts and at desired time points and, further, generate novel structures (Staniek *et al.*, 2013).

Dhurrin, the metabolite in question in the study of Gnanasekaran *et al.* (2016) is a cyanogenic glucoside originating from *Sorghum* spp. Although probably of no direct use to people, it confers enormous advantage to its host plants by deterring feeding insects (Tattersall *et al.*, 2001). For delineation of plant metabolism the pathway leading to dhurrin seems to provide an ideal playground. First, it takes only three enzymatic steps (from tyrosine, the primary metabolism-derived amino acid) to yield the final product, making it a relatively short and straightforward metabolic route. Second, the pathway enzymes have to act in a tightly regulated and choreographed manner. Since intermediates on the way from tyrosine to dhurrin are toxic to the cell, the catalysts need to operate in a kind of ‘bucket chain’, handing over the product of one reaction to the following actor, thereby minimizing leakage and preventing toxicity. While unequivocally demonstrated for dhurrin biosynthesis, the so-called *metabolon* principle is postulated to be relevant to many more pathways (Moller, 2010). Third, the three actors in this short pathway are members of large enzyme families – cytochrome P450 monooxygenases (‘P450s’) and UDP-glucosyl transferases – whose relatives can be found in almost all pathways of plant metabolism. Therefore, the findings of Gnanasekaran *et al*. could possibly be extrapolated to numerous PNP pathways and might help engineer synthetic metabolic pathways for enhanced product formation.

## Versatile biocatalysts

The first two steps of dhurrin biosynthesis are catalyzed by P450s. These are extremely versatile biocatalysts present in all five biological kingdoms. Formally considered monooxygenases, capable of introducing a single oxygen atom into a substrate, they in fact catalyze many more reactions, including oxidations, reductions, dehalogenations, or C–C bond formation. Hence, P450s can introduce various modifications into a given compound skeleton which, very often, makes them key players in a large number of PNP biosynthetic pathways. It is, therefore, not surprising that plant genome investigations have revealed that many species harbor 200–300 different P450s, most of which are thought to be involved in metabolite formation.

To perform their complex reactions, P450s usually need two reducing equivalents from NADH or NADPH, transferred by redox partners, either cytochrome b5 or a specialized P450 reductase. Hence, it has been common sense that one has to provide an appropriate P450 reductase to obtain functional P450 enzymes.

Gnanasekaran *et al.* (2016) have now explored the capacity of chloroplasts to host operational P450s and serve as a framework for metabolite formation. Previous studies indicated that nuclear-encoded P450s can be redirected into chloroplasts and show functionality, but factors like expression level and protein stability seemed to reduce yields of the desired metabolites. Direct transformation of the chloroplast genome might provide an alternative solution. In numerous studies, it has been shown that single transgenes can be functionally expressed from plastid genomes for various purposes (Bock and Warzecha, 2010). More recently, however, Bock and coworkers demonstrated that plastids have the capacity to host and express multiple transgenes, thus providing a blueprint for artificial pathway design and build-up (Lu *et al.*, 2013). Gnanasekaran *et al*. utilized this transgene stacking methodology to introduce all three dhurrin biosynthetic genes into the tobacco plastid genome and were able to show that the recombinant P450s converted tyrosine without a specific redox partner, most probably obtaining electrons directly from photo-reduced ferredoxin. Not only is this the proof that a P450 can reside in the thylakoid membrane (instead of the endoplasmic reticulum) and get electrons directly from light reactions (Box 1), it also asserts the great potential of plastid engineering for the formation of specialized compounds.

**Box 1. Transfer of the dhurrin biosynthetic pathway into chloroplasts**

**Figure:**
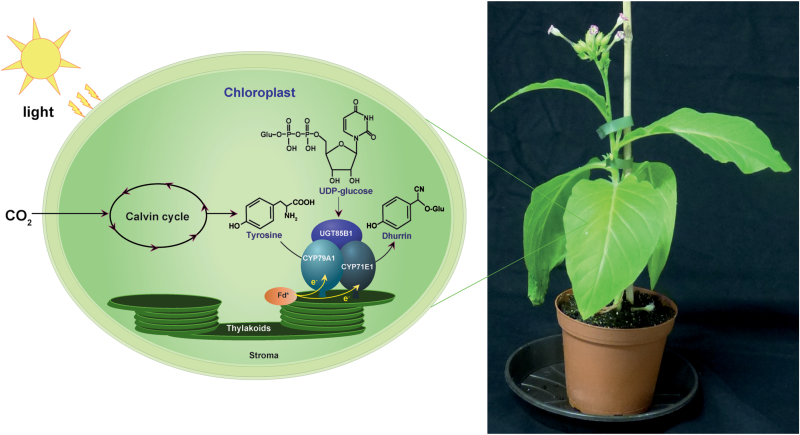
Transfer of the dhurrin biosynthetic pathway from *Sorghum bicolor* comprising membrane-bound cytochrome P450s (CYP79A1 and CYP71E1) and a soluble glucosyltransferase (UGT85B1) into the chloroplasts of *Nicotiana tabacum*. The recombinant P450s are enriched in the thylakoids and most probably receive electrons (e–) directly via photo-reduced ferredoxin (Fd*). Courtesy of Dr Thiyagarajan Gnanasekaran.

## Plastid engineering

While the elucidation of pathways leading to PNPs has greatly accelerated (Staniek *et al.*, 2014), most engineering attempts are made in heterologous hosts, mainly yeast (*Saccharomyces cerevisiae*). Recent reports on the formation of plant compounds like morphinans (Hawkins and Smolke, 2008) or the monoterpenoid indole alkaloid precursor strictosidine (Brown *et al.*, 2015) in yeast highlight the potential this fermentable microorganism might have for the production of PNPs. Inadvertently, however, they also reveal the problems and downsides of the system, pointing especially to the performance of plant P450 enzymes in the heterologous host as the crucial bottleneck. It is not yet clear if NADPH-availability or protein stability and performance are the responsible factors, but the amounts of metabolites retrieved so far fall far below industrial application levels. Engineering plants to be alternative production systems now becomes a valuable recourse, as Gnanasekaran *et al*. have clearly shown that plastid engineering can yield significant amounts of specialized metabolites from pathways involving P450s.

There are, however, some lingering questions to be answered. As to the occasionally negative effects of high transgene expression in plastids – be it due to high amounts of foreign proteins present in the membrane system reducing the electron flow or the leakage of toxic intermediates resulting from ineffective metabolon formation – the transplastomic plants exhibit unfavorable phenotypes and retarded growth (Hennig *et al.*, 2007). Recent advances in engineering of switchable gene expression in plastids will help overcome these obstacles, enabling the tuning of metabolite formation (Emadpour *et al.*, 2015). Further, only three transgenes have been combined in a synthetic operon so far, begging the question of extended transcriptional unit integration into the plastid genome. Again, future developments will show whether it is possible to engineer plastid genomes on a larger or even global scale, generating synthetic genomes for easier manipulation (Scharff and Bock, 2014). Nonetheless, performance of the light-driven P450 catalysts should no longer be the bottleneck.
